# Supplementary material for: CTG-Net: Cross-task guided network for breast ultrasound diagnosis
Source: PLoS One. 2022 Aug 11;17(8):e0271106. doi: 10.1371/journal.pone.0271106 (PMC9371312; doi:10.1371/journal.pone.0271106)
Supplement: Attachment — Submitted filename: Response to Reviewers.pdf [file pone.0271106.s002.pdf]

2022/06/19

Dear Dr. Sathishkumar V E,

My co-authors and I would like to express our gratitude and appreciation for giving us an opportunity to revise our manuscript and for your positive and constructive comments and suggestions on our manuscript titled “CTG-Net: Cross-task guided network for breast ultrasound diagnosis”. The manuscript ID is PONE-D-22-12663.

We have studied the reviewer’s comments carefully and tried our best to revise our manuscript according to these comments. We prepared two parts for the resubmission of our manuscript: (a) our point-by-point response to the comments from reviewers and (b) an updated manuscript labeled “Revised Manuscript with Track Changes”. We believe that our manuscript has considerably improved because of these revisions. Please check the attached manuscript for details.

Each of the authors confirms that this updated manuscript has not been previously published and is not currently under consideration by any other journal. Additionally, all the authors have approved the contents of this paper and have agreed to the *PLOS ONE's* submission policies.

Again, we would like to express our great appreciation to you and the reviewers for your comments and suggestions on our paper. We thank you for the time you put in reviewing our paper and hope the updated version meet your expectations. Looking forward to hearing from you.

Thank you and best regards.

Sincerely,

Graduate School of Science and Technology, University of Tsukuba, Tsukuba, Japan
National Institute of Advanced Industrial Science and Technology, Tsukuba, Japan
Kaiwen Yang
E-mail: kevin.yang@aist.go.jp

Dear Dr. Sathishkumar V E,

First of all, thank you very much for your request for revision related to the journal requirements. Here, we have provided detailed responses to each of these journal requirements and to each reviewer's comments. In addition, we have redone the English correction, and made some minor corrections to paragraphs that the reviewers did not point out without changing the meaning of the text.

1. Please ensure that your manuscript meets PLOS ONE's style requirements, including those for file naming. The PLOS ONE style templates can be found at
https://journals.plos.org/plosone/s/file?id=wjVg/PLOSOne_formatting_sample_main_body.pdf
and
https://journals.plos.org/plosone/s/file?id=ba62/PLOSOne_formatting_sample_title_authors_affiliations.pdf

Authors' Reply: Thank you for providing the style templates for *PLOS ONE*. We have revised the manuscript according to its style requirements.

2. Please amend your current ethics statement to address the following concerns:
a) Did participants provide their written or verbal informed consent to participate in this study?
b) If consent was verbal, please explain i) why written consent was not obtained, ii) how you documented participant consent, and iii) whether the ethics committees/IRB approved this consent procedure.

Authors' Reply: Thank you for your pointing this out. Rest assured that we have conducted an in-depth investigation on this issue and revised the ethics statement in **Dataset subsection of the manuscript (pages 6, lines 199-218)**. The changes are as follows:

{*Since this research does not use biological tissues obtained from the human body, it is not necessary to obtain informed consents from the research participants following the "Ethical Guidelines for Medical Research Involving Human Subjects" set by the Japanese Ministry of Health, Labour and Welfare. Therefore, data were collected using the opt-out method rather than obtaining informed consents from the participants. Specifically, the following information regarding the study was posted on the website of Takamatsu Heiwa Hospital, allowing research participants to refuse the following: (1) outline of the study; (2) names of the research institution, head of the research institution, and principal investigator; (3) statement that the research protocol and materials of this study may be obtained or inspected and the method of obtaining or inspecting such materials (the statement also mentions that the information may not be obtained or viewed if it would interfere with the protection of the personal information or intellectual property of research participants); (4) procedures for disclosure of personal information; (5) notification of the purpose of using personal information and the method of handling personal information, including the fact that participants may refuse to have their personal information provided to outside organizations; and (6) contact information for inquiries and complaints. This procedure was approved by the Ethics Committee of Takamatsu Heiwa Hospital on January 18, 2018, and by the Ethics Committee of the National Institute of Advanced Industrial Science and Technology on March 9, 2018 (No. hi2018-0267).*}

3. Please include information in the Methods section on how the patient data was obtained, and

include information on whether the IRB approved this study, and the name of the IRB. Please also include information on how and when participants provided consent for their data to be used in research."

<u>Authors' Reply:</u> We thank you for pointing out this significant issue regarding the dataset preparation. We agree that it is an important issue, and we apologize that we have not clearly presented the related information. We have updated the collection protocols: (1) selection criteria and (2) exclusion criteria to clarify the patient data in the **Dataset subsection of the manuscript (pages 6-7, lines 233-249)**. Please see the following text.

{*The THH dataset were obtained by breast surgeons with over 17 years of experience using ultrasound equipment from patients who underwent breast ultrasound examinations at Takamatsu Heiwa Hospital between May 2012 and January 2017 and met the following criteria.*

- *Selection criteria: (1) Those with findings, such as masses and nonmassive lesions in the mammary glands, or without apparent findings; and (2) those who do not refuse to participate in this study.*
- *Exclusion criteria: Exclusion criteria: (1) patients who have undergone mastectomy; (2) those with substantially thicker or thinner mammary glands or mammary glands with severe mastopathy; (3) those with inadequate samples; and (4) those who are deemed inappropriate as research participants by the principal investigator.*

This procedure was approved by the Ethics Committee of Takamatsu Heiwa Hospital on January 18, 2018, and by the Ethics Committee of the National Institute of Advanced Industrial Science and Technology on March 9, 2018 (no. hi2018-0267).

The UDIAT and BUSI datasets were provided by [46] and [47], respectively, and were used under institutional or patient approval. Detailed public datasets access information are provided by the Supporting Information S2_data and S3_data.}

4. Please note that PLOS ONE has specific guidelines on code sharing for submissions in which author-generated code underpins the findings in the manuscript. In these cases, all author-generated code must be made available without restrictions upon publication of the work. Please review our guidelines at https://journals.plos.org/plosone/s/materials-and-software-sharing#loc-sharing-code and ensure that your code is shared in a way that follows best practice and facilitates reproducibility and reuse.

<u>Authors' Reply:</u> Thank you for the constructive suggestion regarding code sharing. We agree that reproducibility and replicability are of core significance for scientific publications and source code plays a central role to address ambiguity in the research results. Therefore, we prepared source code with full annotations which are consistent to the flowchart and algorithm explanations presented in the manuscript. For details, please see the zip file in the **Supporting information subsection (page 18, lines 517-520)**.

5. Please review your reference list to ensure that it is complete and correct. If you have cited papers that have been retracted, please include the rationale for doing so in the manuscript text, or remove these references and replace them with relevant current references. Any changes to the reference list should be mentioned in the rebuttal letter that accompanies your revised manuscript. If you need to cite a retracted article, indicate the article's retracted status in the References list and also include a citation and full reference for the retraction notice.

Authors' Reply: Thank you for the comments to the reference section. We have carefully checked the reference list and updated it to ensure its completeness and correctness. We have highlighted these additions in the **References section** of the revised manuscript **(pages 18-23)**.

The following are the additions to the literature:

{References 14, 15, 18, 19, 21, 27-33, and 36-40}

Dear reviewer #1,

Thank you for your valuable advice. After carefully studying your comment, we have finished the revised manuscript and labeled 'Revised Manuscript with Track Changes'. Below is a description of the point-to-point changes we have made based on your comments.

Comment 1. Add in the last lines of Abstract in what %age and in what parameters, the proposed methodology is better as compared to existing techniques and what is the overall analysis of the proposed methodology.

Authors' Reply: According to your comments regarding quantitative evaluation, we have revised the content of {*We performed extensive experimental comparison on both private and public ultrasound datasets and the results validated that the proposed approach achieved significant improvements compared with the state-of-the-art methods. The results demonstrate the effectiveness of the proposed cross-task guided feature learning framework for ultrasound image recognition, and it can be one potential solution for clinical application in aiding sonographers to detect and diagnose breast cancer.*} in the previous manuscript to

{*We performed extensive experimental comparisons on multiple ultrasound datasets. Compared to state-of-the-art multi-task learning approaches, the proposed approach can improve the Dice's coefficient, true positive rate of segmentation, AUC, and sensitivity of classification by 11%, 17%, 2%, and 6%, respectively. The results demonstrate that the proposed cross-task guided feature learning framework can effectively fuse the complementary information of ultrasound image segmentation and classification tasks to achieve accurate tumor localization. Thus, it can aid sonographers to detect and diagnose breast cancer.*} **(Abstract section, page 1)**.

Comment 2. Under contributions, add one-two points with regard to experimentation. Add Organization of the paper at end of introduction.

Authors' Reply: For the top level design of our experimental validation, we mainly addressed two points: 1. We do fair comparison with other latest MTL methods (**page 3, lines 78-80)**. 2. The tests were performed upon multiple datasets and thus we avoid data-induced bias in performance comparison (**page 3, lines 81-83)**. Furthermore, we presented all the details of experiments in revised manuscript, including dataset (**pages 5-7, lines 189-249)**, parameter settings (**page 11, lines 353-365)**, evaluation protocol (**page 11, lines 366-372)** and ablation study scheme (**pages 16-17, lines 441-494)**. From fundamental design to implementation details, we organized those contents in a logical manner to ease understanding.

Specifically, we have added one point with regard to experimentation under contributions (**page 3, lines 81-83)**. The additions in the revised manuscript are as follows:

{

- *The proposed approach achieves excellent performance on several private and public datasets with visual differences proving that the proposed approach has good generalization performance and can minimize bias caused by the dataset.*}

In addition, we have also added the organization of this paper at the end of the introduction (**page 3, lines 84-89**), please see as follows:

{*The rest of the paper is organized as follows. Section 2 introduces the related work to the proposed method, Section 3 describes the datasets adopted and explains the overall structure of the proposed*

method, component units, and loss function. Section 4 presents the experimental setup, evaluation metrics, and experimental results. Section 5 discusses the ablation experiments and failure cases. Finally, this study is concluded in Section 6.}

Comment 3. Under literature review, it is suggested to add min 15-25 papers which are latest and taken as base for the proposal of methodology, and every paper should be elaborated with what is proposed, what is the novelty and what experimental results are there. At the end of Literature review, highlight in 9-15 lines what overall technical gaps are there in the paper, that led to the design of proposed methodology.

Authors' Reply: According to your comments regarding the literature review, we have made substantial revision to the current reference list, and the current version covers representative methods for BUS image analysis, some of which are used as baselines for experimental comparisons. Concretely, we have added more recent literature in accordance with your comment. These are the literature recommended in comment 9 and the latest literature about multi-task learning.

The following are the additions to the literature:

References 14, 15, 18, 19, 21, 27 in **page 4, lines 111-120.**

References 28-33 in **page 4, lines 136-140.**

{*With the idea of MTL being widely investigated in natural images [28-30] and other types of medical images (dermoscopy color images [31], abdominal computed tomography scans [32], brain magnetic resonance images [33]), jointly trained BUS image classification and segmentation has also evolved as a major topic.*}

References 36-38 in **pages 4-5, lines 145-156.**

{*Zhou et al. [36] used the VNet architecture to develop a CAD system that can jointly perform 3D automatic breast ultrasound (ABUS) image classification and segmentation CAD system. They exploited the extracted multi-scale features to improve the image classification task and achieve better results than a single task through an iterative feature refinement strategy. Zhang et al. [37] proposed BI-RADS-Net for explainable BUS CAD based on multi-task learning. The model outputs the probability of class and malignancy of a tumor by performing multiple classification and regression tasks. Cao et al. [38] proposed a multi-task learning method based on label distribution correction for overcoming the problem of insufficient labeled training data. They performed breast tumor classification task jointly using two labels from different domains of expertise and demonstrated the effectiveness of the method on the collected dataset.*}

In addition, based on the literature review, we have provided the following analysis to illustrate the advantages of the proposed method.

{*The above survey reveals that the MTL approach could be a promising approach, however, there was little in-depth investigation along this research direction. In contrast to previous studies, our contribution is three-fold, which are as follows. First, it is acknowledged that finding suitable auxiliary tasks plays the most important role for MTL. The tasks should have some level of correlation, otherwise, training on irrelevant tasks can result in negative transfer and deteriorate the performance. To the best of our knowledge, this is the first study to formulate lesion classification and its region segmentation as a multi-task learning problem for BUS image analysis. The two tasks are highly correlated and thus appropriate to be investigated through multi-task learning.*

Second, to achieve superior performance in lesion classification and segmentation, we adopted the attention mechanism in the proposed neural network design, which enables the network to focus on

a few particular aspects that are related to suspicious lesion areas and ignore the rest. In other words, it is an integral building block to generating pixel-wise labels for the lesion region.
Third, MTL has been commonly formulated as a minimization of a linear combination of individual tasks' loss functions. The task-specific weights are critical parameters to tune through the learning process. We adopted a self-adjusted scheme to estimate the task-specific weights through optimization, which is more efficient and robust compared to conventional methods such as grid search through cross-validation.} **(page 5, lines 157-175)**

Comment 4. Under methods, add the methods, which are used to design the proposed methodology.
Authors' Reply: We have checked the manuscript carefully and added further details regarding the design of the proposed method **(page 7, lines 258-265)**. The added materials illustrate how our proposed approach ensures that segmentation and classification can be effectively facilitated between them.
{*Classification and segmentation can achieve mutual complementarity based on two critical evidences: (1) Previous studies [7][8] demonstrated that segmentation can provide classification with prior lesion localization information and help classification to exclude interference from regions that hinder judgment. (2) Previous studies [39][40] performed weakly supervised segmentation using class activation maps for classification demonstrated that class-specific diagnostic information can highlight lesion regions to help fine segmentation.*}

work [7]: Huang, Y, Han L, Dou H, Luo H, Yuan Z, Liu Q, Zhang Z, Yin G. Two-stage CNNs for computerized BI-RADS categorization in breast ultrasound images. Biomed. Eng. Online. 2019;18(1): 1-18.

work [8]: Wang, F, Liu X, Yuan N, Qian B, Ruan L, Yin C, Jin C. Study on automatic detection and classification of breast nodule using deep convolutional neural network system. J. Thorac. Dis. 2020;12(9):4690.

work [39]: Huang, Y, Chung A. Evidence localization for pathology images using weakly supervised learning. International Conference on Medical Image Computing and Computer-Assisted Intervention. Springer, Cham, 2019.

work [40]: Liang, G, Wang X, Zhang Y, and Jacobs N. Weakly-supervised self-training for breast cancer localization. 2020 42nd Annual International Conference of the IEEE Engineering in Medicine & Biology Society (EMBC). IEEE, 2020.

Comment 5. Add proper system model of the proposed methodology. Add algorithm and flowchart of the proposed methodology
Authors' Reply: According to your suggestions regarding the proposed methodology, we revised our paper as follows. In the **Methodology section (from page 7)** of the manuscript, we have described the system model of the proposed method in detail. Moreover, the flowchart of the proposed methodology has been presented in **Fig. 5**. We have added the algorithmic pseudo-code **(page 12)** for model optimization and modified the manuscript **(page 11, lines 364-365)** below to further illustrate the approach in this study.
{*Algorithm 1 provides the algorithm details to clearly show the optimization process of our proposed method.*}
We believe that Algorithm 1 clarifies doubts regarding the algorithm implementation.
The following figure shows Algorithm 1.

Algorithm 1 Cross-task guided network CTG-Net

Input: BUS image I with segmentation true mask and class true label
Initialization: Feature extraction unit use VGG16 pre-trained with ImageNet, other parts use random values
repeat
  $F' \leftarrow$ ASPP(pre-trained VGG16(I)) ▷ ASPP: atrous spatial pyramid pooling
  $M(M_l, M_m) \leftarrow \text{softmax}(\text{upsample}(F'))$
  $G_{\text{global}} \leftarrow \text{FCLayer}(\text{GAP}(\text{LAM}(F', M)))$ ▷ GAP: global average pooling
  $H_{\text{score}} \leftarrow \text{softmax}(G_{\text{global}})$
  $F_{\text{fine}} \leftarrow \text{Concat}(\text{CSM}(F', H_{\text{score}}, G_{\text{global}}), \text{AKGM}(F', M_{\text{m}}))$
  ▷ Concat: concatenation operation
  Fine mask $\leftarrow \text{softmax}(\text{upsample}(F_{\text{fine}}))$
  $\ell_{\text{cseg}}, \ell_{\text{cls}}, \ell_{\text{fseg}} \leftarrow$ calculate the respective losses using $\{M, H_{\text{score}}, \text{Fine mask}\}$
  Calculate ℓ_{total} by Eqs. (8) and (9)
  Update model parameter θ by Adam optimizer
until validation set not improve after 10 consecutive epochs
Output: Parameter set of the CTG-Net model θ
function1 LAM(F': shared features, M: coarse mask ($M_{\text{l}}, M_{\text{m}}$))
  $F_{\text{ma}} \leftarrow F' \odot M_{\text{m}}$
  $F_{\text{la}} \leftarrow F' \odot M_{\text{l}}$ ▷ $\odot$: element-wise multiplication
  Return: $F_{\text{LAM}} \leftarrow F' + F_{\text{ma}} + F_{\text{la}}$
end function1
function2 CSM(F', H_{score}: prediction scores, G_{global}: global feature vector)
  Return: $F_{\text{CSM}} \leftarrow [\text{Conv}(G_{\text{global}}) \odot H^T_{\text{score}}]\ \text{Conv}(F')$
  ▷ Conv: convolutional layer operations
end function2
function3 AKGM(F', M_{m}: mammary gland map)
  Calculate S using $\{M_{\text{m}}, F'\}$ by Eq. (6) ▷ S: similarity weights
  $V \leftarrow \text{Conv}(F')$
  Return: $F_{\text{AKGM}} \leftarrow \alpha(S \otimes V) + F'$
  ▷ α: learnable parameters, $\otimes$: matrix multiplication
end function3

Comment 6. Add Analysis section to the paper.

Authors' Reply: We apologize for the unclear expression due to writing problems. To clearly explain the analysis section in our manuscript, we have revised the text related to the analysis in the revised manuscript.

The following are the contents related to the analysis.

1) Comparative experimental analysis to previous methods. We conducted extensive comparative experiments with state-of-the-art segmentation, classification, and multi-task learning methods to analyze the superiority of the proposed method. We have modified the grammatical writing of this part to make it easier to understand. Detailed experimental results and specific analysis are presented in the **Comparisons with state-of-the-art methods subsection (pages 13-15, lines 375-439)**.
2) Error analysis and discussion for future improvement. To analyze the limitations of the proposed method, we have performed a detailed analysis based on some cases of recognition failure (**Limitations subsection, page 17, lines 481-489**), and added the limitations of the proposed method (**Limitations subsection, page 17, lines 490-493**). In addition, we also added directions for future work based on potential solutions (**at the end of the Conclusion section, page 18, lines 507-511**).
3) Overall performance. We performed an exhaustive ablation experiment to analyze the

effectiveness of our proposed overall structure and individual modules in **Discussions section**. For example, as discussed in the **Ablation study subsection (page 16, lines 444-449)**, we compared and analyzed the overall performance of the network without using any module to the proposed method.

Comment 7. Add some case study based discussion to the paper.

Authors' Reply: We have accordingly presented the discussed cases from several aspects and modified the grammatical writing to further facilitate understanding.

1) We have revised the **Comparisons with state-of-the-art methods section** of our manuscript, and selected cases for discussion in the private dataset **(pages 13-14, lines 385-388, 395-399** illustrated in **Fig. 9)** and public datasets **(page 14, lines 412-420** illustrated in **Fig. 10)**, respectively. These case studies show the advantages of the proposed method over other state-of-the-art methods.
2) We have discussed in detail the four failed cases in F**ig. 11** and further added the limitations of this study (**Limitations subsection, pages 17, lines 490-493**).

Comment 8. Add future scope to the paper.

Authors' Reply: We agree that adding the future scope will make this study more complete. We have added the future work **at the end of the Conclusion section (page 18, lines 507-511)** in the revised manuscript. The following are the additions:

{*In future work, we will investigate more efficient loss functions to help the proposed model obtain more robust BUS diagnosis results. This includes the introduction of a boundary loss function to help obtain explicit boundaries for different lesions and the investigation of a loss function to help obtain uniform recognition results to ensure consistency of prediction in segmentation and classification.*}

Comment 9. Considering the scope of the paper, add the following references and highlight them properly in the manuscript:

a. Ajantha Devi, V., & Nayyar, A. (2021). Fusion of deep learning and image processing techniques for breast cancer diagnosis. In Deep learning for cancer diagnosis (pp. 1-25). Springer, Singapore.

b. Solanki, A., & Nayyar, A. (2020, December). Transfer Learning to Improve Breast Cancer Detection on Unannotated Screening Mammography. In International Conference on Advanced Informatics for Computing Research (pp. 563-576). Springer, Singapore.

Authors' Reply: We have added the recommended works in the **Related Work section** of the revised manuscript. Please see **references 15 and 18**. The additions in the revised manuscript are as follows:

{*such as combining DCNNs with image processing techniques to help classification [15]. The latest tweaks to neural networks, such as deep transfer learning [16-19] and the attention model, have also been employed.*} **(page 4, lines 112-114)**

Dear reviewer #2,

Thank you for your thoughtful review comments and questions that help us improve our manuscripts. We have addressed all the issues in the file labeled 'Revised Manuscript with Track Changes'. Here below is the description of the revisions made according to your comments.

Comment 1. Why Adam optimizer is used?

Authors' Reply: There is no definite conclusion on which optimizer should be used in deep learning model training. Adam is used in this study for the following two reasons:

(1) Adam has the advantages of adaptive learning rate, and simple and efficient implementation, and it is one of the most commonly used optimizers. Furthermore, this study [1] demonstrated that ADAM can be generally better than SGD.

(2) In previous experiments, we used Adam, SGD, and Adagrad optimizers for comparison, and the final results show that Adam performs better for this task.

[1] Choi, Dami, et al. "On empirical comparisons of optimizers for deep learning." arXiv preprint arXiv:1910.05446 (2019).

We mentioned content related to this comment in the **Experiment setup subsection (page 11, lines 357-361)**. The additions in the revised manuscript are as follows:

{*Although better optimization methods have been proposed, the Adam optimizer has a simple mechanism and is often used as a standard optimization method by many methods. Therefore, this study uses the Adam optimizer to assess the intrinsic superiority of the proposed approach by checking whether the standard optimization method can also obtain good performance.*}

Comment 2. References should be updated with recent works related to the proposed study

Authors' Reply: We have updated more recent literature based on your comments. The following is the list of the added literature:

{References 14, 15, 18, 19, 21, 27-33, and 36-40}

The following is a detailed description:

- References 14, 15, 18, 19, 21, 27 in **page 4, lines 111-120.**
- References 28-33 in **page 4, lines 136-140.**

{*With the idea of MTL being widely investigated in natural images [28-30] and other types of medical images (dermoscopy color images [31], abdominal computed tomography scans [32], brain magnetic resonance images [33]), jointly trained BUS image classification and segmentation has also evolved as a major topic.*}

- References 36-38 in **pages 4-5, lines 145-156.**

{*Zhou et al. [36] used the VNet architecture to develop a CAD system that can jointly perform 3D automatic breast ultrasound (ABUS) image classification and segmentation CAD system. They exploited the extracted multi-scale features to improve the image classification task and achieve better results than a single task through an iterative feature refinement strategy. Zhang et al. [37] proposed BI-RADS-Net for explainable BUS CAD based on multi-task learning. The model outputs the probability of class and malignancy of a tumor by performing multiple classification and regression tasks. Cao et al. [38] proposed a multi-task learning method based on label distribution correction for overcoming the problem of insufficient labeled training data. They performed breast tumor classification task jointly using two labels from different domains of expertise and*

demonstrated the effectiveness of the method on the collected dataset.}

References 39-40 in **page 7, lines 262-265.**

{*Previous studies [39][40] performed weakly supervised segmentation using class activation maps for classification and demonstrated that class-specific diagnostic information can highlight lesion regions to help fine segmentation.*}

Comment 3. Include the limitations of the study

Authors' Reply: In the revised manuscript, we have changed the **sub-title "Failure case visualization"** to **"Limitations"**. We further analyzed the limitations of this study **(page 17, lines 490-493)**. The following are the additions in the revised manuscript:

{*The problem of blurred boundaries in few samples remains challenging. Therefore, we intend to introduce the loss of lesion boundaries to address this problem. In addition, for the problem of inconsistent predictions of segmentation and classification tasks, an effective loss function should be designed to supervise each other's inter-task outputs and ensure the consistency of their predictions.*}

Comment 4. Future work should be added in the conclusion section

Authors' Reply: From your comments, we realized that the future scope is an integral part. In the revised manuscript, we have added the future work at the end of the **Conclusion section (page 18, lines 507-511)**. The following are the changes made:

{*In future work, we will investigate more efficient loss functions to help the proposed model obtain more robust BUS diagnosis results. This includes the introduction of a boundary loss function to help obtain explicit boundaries for different lesions and the investigation of a loss function to help obtain uniform recognition results to ensure consistency of prediction in segmentation and classification.*}

Comment 5. Provide explanation for wi used in equation 9

Authors' Reply: We have added the explanation for w_i used in Eq. (9) to address your concerns. Please see the **Loss function subsection** of the manuscript **(page 11, lines 345-346, 348-349)**.

The following are the changes made:

{i *is the number of tasks,* $w_i(\cdot)$ *calculates the relative descending rate of loss for each task. and* $L_k(\cdot)$ *is the loss value for each iteration.*}

Comment 6. Why 5 fold cross validation is used?

Authors' Reply: This is indeed a crucial point regarding experimental validation protocols. To the best of our knowledge, there is no absolute conclusion on the specific choice of how many folds to use for cross-validation in the research field of computer vision-based BUS image analysis.

This study used five-fold cross-validation due to the following reasons:

(1) According to literature review, it is the most applied validation protocol for empirical analysis. Thus, we adopt same criterion to maximize a fair comparison.

(2) Five-fold is chosen as a solution after balancing the evaluation of the model performance with the computational cost. For example, the training set is closer to the real sample space, and the bias in the final evaluation of the model is reduced when a fold is chosen for a large number. However, the computational cost increases accordingly.

We mentioned the content related to this comment in the **Dataset subsection (pages 5-6, lines 194-197)**. The following are the changes made:

{*We conducted experiments using fivefold cross-validation, which is the most commonly applied validation protocol for empirical analysis, on each of the three datasets. Therefore, we adopted the same criterion to maximize a fair comparison.*}

Comment 7. Any hyperparameters associated with the models used in this study?

Authors' Reply: We apologize for the unclear expression due to writing problems. We have revised the text to make it easier to understand. The changes made in the revised manuscript are as follows:

(Loss function subsection, pages 10-11, lines 339-349)

(Experiment setup subsection, page 11, lines 353-365)

In addition, we have listed the following hyperparameters involved in this study. All of these hyperparameters are mentioned in the manuscript.

1. *Initial learning rate: 0.0001* **(Experiment setup subsection, page 11, line 357)**
2. *epochs: 100* **(Experiment setup subsection, page 11, line 362)**
3. *batch-size: 16* **(Experiment setup subsection, page 11, line 362)**
4. *activation function: Softmax function* **(Lesion classification unit subsection, page 9, line 319)**
5. *optimizer: Adam (β_1=0.9, β_2=0.99, ϵ=1e-08)* **(Experiment setup subsection, page 11, lines 356-357)**
6. *Multi-task loss function weights:* **(Loss function subsection, pages 10-11, lines 339-349)**

$$L_{\text{total}} = \lambda_1 L_{\text{cls}} + \lambda_2 (L_{\text{cseg}} + L_{\text{fseg}}), \tag{8}$$

In Equation (8), λ_1 *and* λ_2 *are the task weights* for the classification and those for the segmentation, respectively. They are calculated using the dynamic weight averaging method.

$$\lambda_k(t) := \frac{K \exp\left(w_k(t-1)/T\right)}{\sum_i \exp\left(w_i(t-1)/T\right)}, \quad w_k(t-1) = \frac{L_k(t-1)}{L_k(t-2)}, \tag{9}$$

In Equation (9), T is a constant for controlling the softness of the task weighting, and T is set to two in this study according to experience and experiments.

Comment 8. What audience would benefit most from this work?

Authors' Reply: First, we consider that sonographers would benefit most from this work. It is because this work has been extensively experimented on breast cancer ultrasound datasets and has yielded positive results.

Second, physicians who use other types of medical images for diagnosis and workers who use image recognition technology to assist in automated production when they have similar tasks are also expected to benefit from this work.

Third, this work demonstrates that using multi-task learning methods to analyze breast ultrasound images is a prospective research topic, and thus, this work could contribute to more in-depth exploration by relevant researchers.

In the revised manuscript., we refer this comment in **page 18, lines 505-506**. The following are the contents:

{*It is anticipated that CTG-Net will improve the accuracy and efficiency of diagnosis by*

sonographers.}

Comment 9. What are the three strongest aspects of this manuscript?

<u>Authors' Reply:</u> We will clarify again the major contributions of this study. We summarize the highlights as follows:

- An individual algorithm for separate BUS image analysis can be aggregated to fuse complementary information for better cognition medical image pattern recognition.
- Attention mechanism brought significant performance gain to general machine learning systems. In this study, we developed a well-designed approach to incorporate attention mechanism with a multi-task learning scheme.
- We performed multi-dataset evaluation and compared the proposed approach with other latest methods for BUS image recognition. The evaluation protocol can minimalize dataset-induced bias and demonstrate the practical performance of algorithms.
